# Use of an External Ventricular Drain for Treatment of a Thoracolumbar Cerebrospinal Fluid Leak: A Case Report and Review of Literature

**DOI:** 10.7759/cureus.24066

**Published:** 2022-04-12

**Authors:** Roberto J Perez-Roman, Jean-Paul Bryant, Harold J Tapamo, Evan Luther, Howard B Levene

**Affiliations:** 1 Neurological Surgery, University of Miami Miller School of Medicine, Miami, USA; 2 Neurological Surgery, Levene Neurosurgical Consulting Inc., Boca Raton, USA

**Keywords:** computed tomography (ct ), subarachnoid hemmorhage, external ventricular drain, csf leakage, cerebrospinal fluid (csf)

## Abstract

Post-operative cerebrospinal fluid (CSF) leak is a known complication in spine surgery. This mostly iatrogenic issue is typically treated using a variety of modalities (i.e., bed rest, epidural patch), CSF diversion methods, or primary repair. The use of an external ventricular drain to treat this post-operative complication has been infrequently reported. We describe a case of a CSF leak after thoraco-lumbar surgery treated using an external ventricular drain and a review of the literature regarding this treatment modality. A 70-year-old man presented to our clinic with a recent diagnosis of multiple myeloma with progressive thoracic kyphosis and spinal stenosis. He developed progressive neurological deficits over the course of several weeks. Radiological studies showed significant thoracic kyphosis and severe cord compression in the thoraco-lumbar area. The patient underwent a T9-L4 posterior instrumentation and fusion with decompression surgery that developed post-operative wound infection and a CSF leak. An external ventricular drain (EVD) was used successfully as a CSF diversion method where direct thoracolumbar approaches were not feasible. Given the effectiveness of EVD placement in treating this post-operative complication, we concluded that the use of an EVD can be a potentially safe and effective way to treat thoracolumbar CSF leakage when lumbar or cervical drainage is not feasible.

## Introduction

Iatrogenic cerebrospinal fluid (CSF) leakage is a common concern in spinal surgery following any procedure involving this region [1]. It is estimated that the overall incidence of dural tear after lumbar decompression is around seven percent [2]. Treatment of an iatrogenic spinal CSF leak involves an array of modalities which includes conservative management with bed rest, use of an epidural blood patch [3], or placement of a lumbar or cervical subarachnoid drain. Drain placement provides a path of least resistance for CSF to divert and allows the site of the dural injury to heal effectively [4]. Ultimately, fibrin glue or tissue grafting with direct closure of the leakage site may be needed [5-6]. The use of an external ventricular drain (EVD) as a CSF diversion method for spinal CSF leak is an infrequently used and rarely reported alternative method in patients who are not candidates for classic CSF diversion techniques [7].

We present a case of a 70-year-old man who underwent T9-L4 posterior instrumentation and fusion surgery and later developed a post-operative CSF leak. The patient subsequently developed a wound infection and was treated successfully with an EVD.

## Case presentation

**Figure 1:**
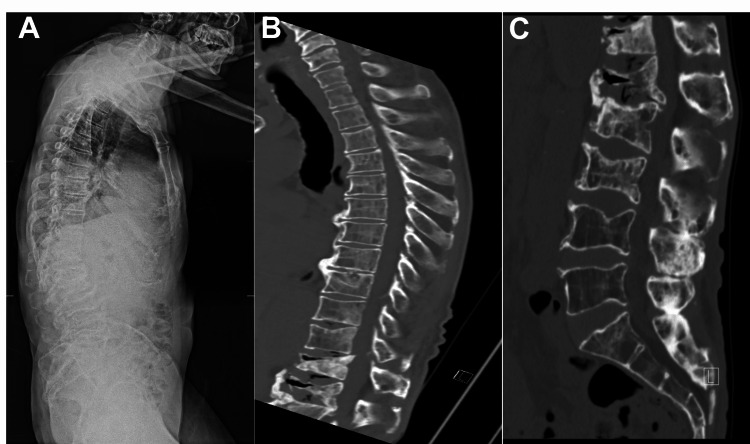
Scoliosis standing X-ray shows significant positive balance with pronounced thoracic kyphosis (A). Sagittal CT without contrast show T12, L1, L2, and L3 compression fractures with severe height loss seen at the L1 body (B & C).

**Figure 2:**
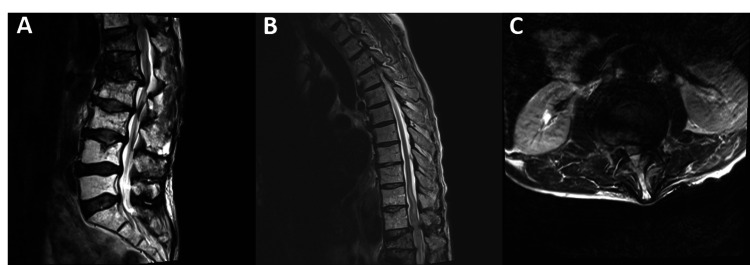
Sagittal (A & B) and axial (C) pre-operative MRI show severe compression at the level of T12 and L1.

A 70-year-old man presented to our clinic with a recent diagnosis of multiple myeloma with progressive thoracic kyphosis. He developed two weeks of bowel and bladder incontinence along with increased difficulty ambulating over the course of several months. On physical exam, the patient had marked distal lower extremity weakness and no rectal tone. Radiological studies showed significant thoracic kyphosis (Figure 1) and severe cord compression at T12- L1 with compression fractures from T12-L3 (Figure 2). 

**Figure 3:**
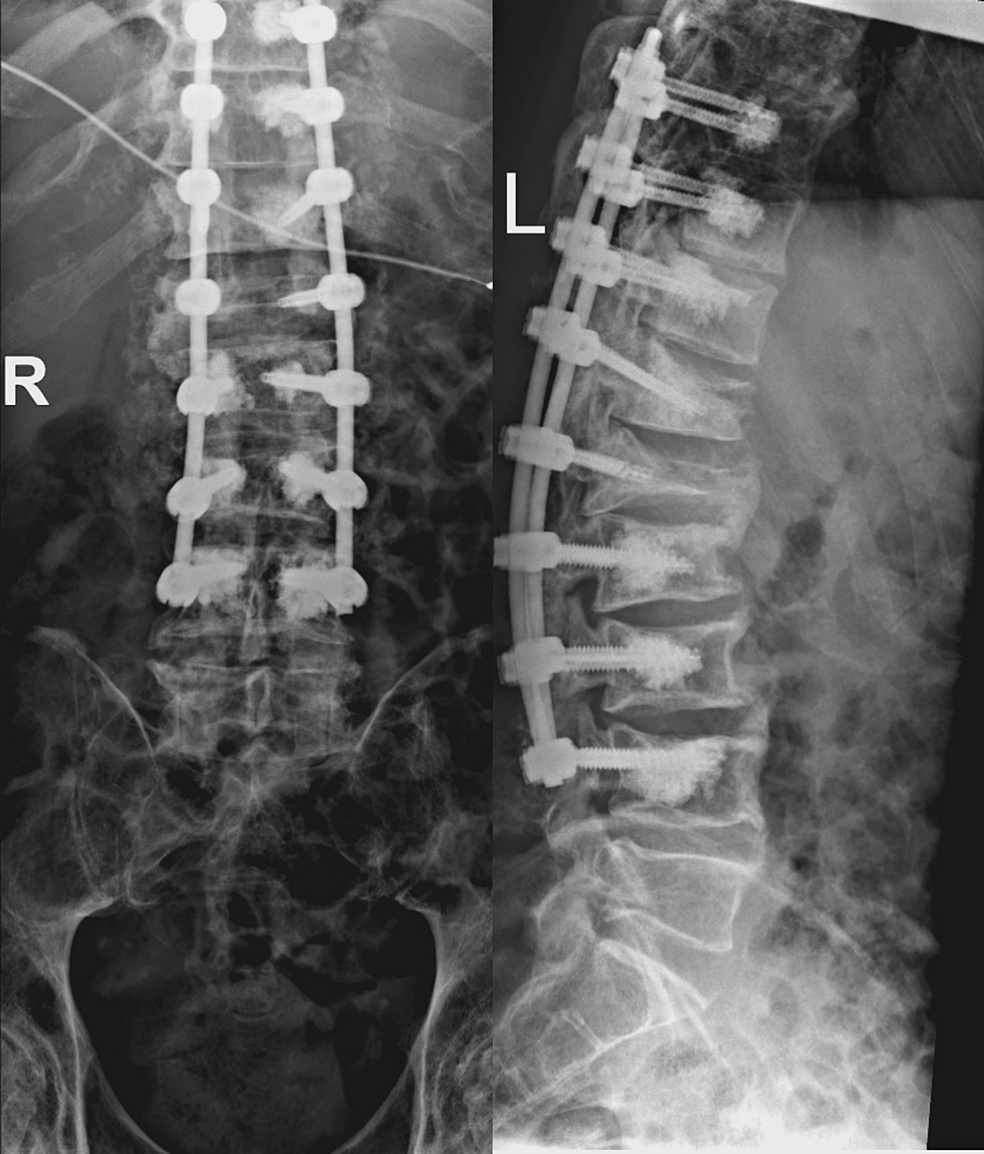
Post-operative X-rays show well-seated T9-L4 posterolateral instrumentation with cement augmentation.

Because of progressive neurological symptoms along with the radiological findings, the patient underwent T9-L4 posterolateral fusion with screw cement augmentation with focal decompression at T11-T12, T12-L1, and L1-L2 (Figure 3). 

**Figure 4:**
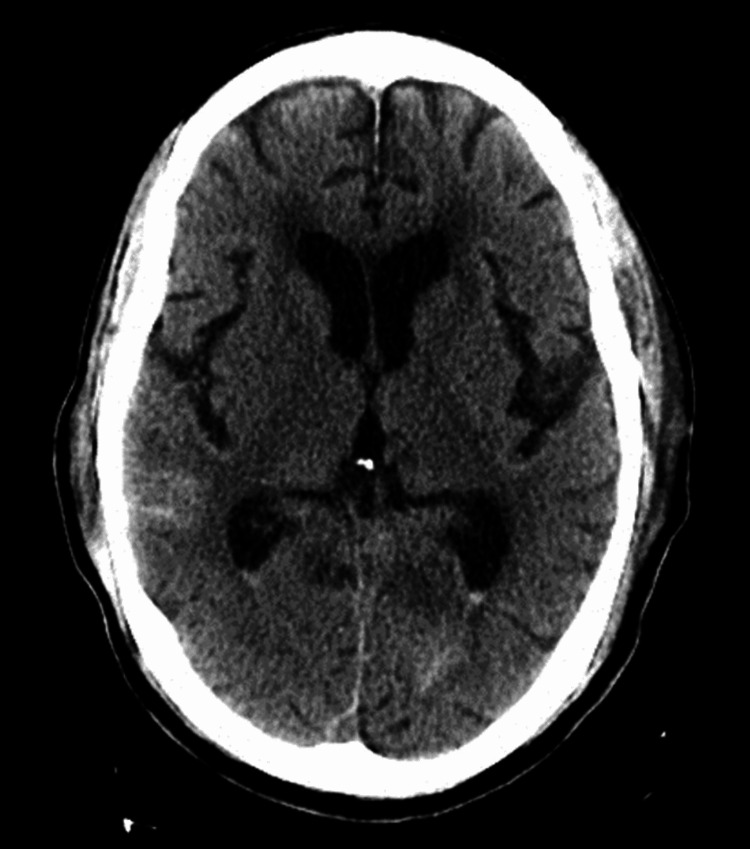
Post-operative CT of the brain without contrast shows scattered subarachnoid blood with some layering on the lateral ventricles along with small amounts of pneumocephalus.

The surgery proceeded without complication. The night following surgery, the patient was noted to be confused with altered mental status. A head CT scan (Figure 4) was performed which showed multifocal subarachnoid hemorrhage (SAH), layering of acute blood in bilateral ventricles, and small amounts of pneumocephalus. 

Discussion with the family and confirmation by urine testing revealed the patient had used cocaine immediately preoperatively which had not been previously disclosed. Symptoms resolved on post-operative day 2 with observation and return to his neurological baseline. On the sixth day following surgery, the incision was inspected and was found to be leaking a non-purulent clear fluid from the inferior aspect. The presence of recent surgery, small amounts of pneumocephalus on post-operative brain imaging, and clear fluid raised concern for a CSF leak. The fluid was sent for beta-2 transferrin testing which came back positive, confirming CSF leakage.

**Figure 5:**
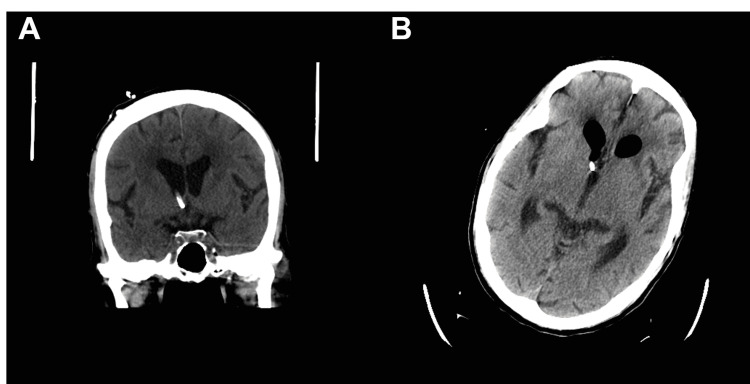
Coronal (A) and axial (B) CT of the brain without contrast showing proper placement of an external ventricular drain.

Due to a large recent thoracolumbar surgery with evidence of lumbar wound dehiscence/infection, a lumbar drain was not feasible as a CSF diversion method. Further, the patient’s clinical status continued to worsen rapidly, suggesting a septic clinical picture. Specifically, the patient's clinical status suggested urosepsis which was immediately treated with broad-spectrum antibiotics. However, acutely, the patient’s status became critical and was subsequently evaluated by palliative care for the potential withdrawal of treatment. Additional imaging studies were desired in order to clearly elucidate the exact site of the CSF leak, however, the patient’s clinical status continued to deteriorate, and an urgent procedure was needed. Given that the patient had become systemically ill, a larger open lumbar surgical exploration and repair was not possible due to the patient’s unlikely ability to tolerate the larger procedure. The authors were aware of diversion techniques involving the placement of a cervical CSF diverting drain [8], however, the institution where the patient was admitted did not offer this technique and he could not be transferred. Further, less invasive procedures such as an epidural blood patch were considered but the leak remained far too copious for this intervention. We decided to proceed with a right external ventricular drain (Figure 5) as a method for CSF diversion.

The patient drained approximately 10 cc of CSF per hour for seven days and the CSF leak was resolved. A three-day clamp trial was done with no further signs of spinal fluid leakage. The EVD was removed and the patient was discharged to the Spinal Cord Injury Rehabilitation unit with no subsequent complications related to CSF leakage.

## Discussion

While primary dural closure and the use of lumbar or cervical drains are common ways to treat thoracolumbar CSF leakage, there are no established guidelines on how to deal with this post-operative complication. It is generally agreed upon that closing the dura primarily in a “watertight” manner when a leak is noted intra-operatively along with post-operative bed rest should be the first step to treat a CSF leak [4]. In a study of dural closure after lumbar surgery, 97.7% of patients (n=88) experienced no further leakage [9]. In the case of our patient, there was no evident dural injury at the time of surgery. Other studies have demonstrated that the use of lumbar subarachnoid drains is an effective method to treat CSF leaks [10]. In our case, a lumbar drain was not placed because of wound dehiscence and infection preventing its placement. The patient was becoming increasingly systemically ill and a larger wound exploration repair would have been considered a high-risk procedure. A drain in the cervical region was not available at the institution where the patient was admitted.

We proceeded with an EVD as a CSF diversion method to allow the site of dural injury to heal. There are few reports of an external ventricular drain used to treat thoracolumbar CSF leakage in the literature. In a study published by Galgano et al., an EVD successfully treated an individual who underwent multiple surgeries for a recurrent ependymoma that developed persistent CSF leakage [7]. Yeager described a case of a patient with a cervical schwannoma who developed a persistent post-operative CSF leak which, after multiple attempts for primary repair and placement of a lumbar subarachnoid drain, only resolved after placement of an EVD with subsequent conversion to a permanent shunt [11].

Our case adds to the experiences reported in the literature to support the use of an EVD as a CSF diversion method to treat complex CSF leaks after spinal procedures in patients who are not candidates for cervical or lumbar cerebrospinal fluid drains. A larger prospective study could elucidate its safety and effectiveness over a long-term period.

## Conclusions

The use of an EVD to treat CSF leaks following spinal procedures is infrequently reported in the literature. In our case study, the patient underwent CSF drainage for seven days and was subsequently discharged with no further complications. Although a CSF leak was not originally avoided, the subsequent diversion method proved to be effective in mitigating this common post-operative complication. While this only represents one case, our favorable outcome suggests that neurosurgeons may consider the use of an EVD as a safe option to treat thoracolumbar CSF leakage when more traditional methods are not feasible.
